# Molecular epidemiology and mechanism of *Klebsiella pneumoniae* resistance to ertapenem but not to other carbapenems in China

**DOI:** 10.3389/fmicb.2022.974990

**Published:** 2022-11-08

**Authors:** Dongliang Wang, Minggui Wang, Tianpeng He, Dan Li, Liqin Zhang, Dongquan Zhang, Junshuai Feng, Wenli Yang, Yuan Yuan

**Affiliations:** ^1^Department of Critical Care Medicine, Gansu Provincial Hospital, Lanzhou, Gansu, China; ^2^Institute of Antibiotics, Huashan Hospital, Fudan University, Shanghai, China; ^3^Key Laboratory of Clinical Pharmacology of Antibiotics, National Health Commission of the People’s Republic of China, Shanghai, China

**Keywords:** ertapenem resistance, *Klebsiella pneumoniae*, *ramR*, efflux pump, outer membrane protein

## Abstract

Resistance to only ertapenem is an unusual phenotype of carbapenem-resistant *Klebsiella pneumoniae* (CRKP). This study aimed to investigate the molecular epidemiology and underlying mechanism involved in ertapenem resistance of *K. pneumoniae* strains that are susceptible to meropenem and imipenem. Among the 697 *K. pneumoniae* strains isolated from 11 grade A hospitals in China, 245 were CRKP strains, of which 18 strains resistant only to ertapenem were isolated. The genotypes, phenotypes, drug resistance homology, and drug sensitivity were analyzed; moreover, the expressions of efflux pump components and outer membrane proteins were assessed. The whole genomes of these 18 strains were sequenced and analyzed for mutations leading to drug resistance. The results revealed that ertapenem resistance may be related to *ramR* mutation. The function of *ramR* was confirmed using gene complementation to the original strain to determine the mechanism underlying ertapenem resistance of *K. pneumoniae* strains. In total, 7.4% of the tested CRKP strains were resistant only to ertapenem. None of these strains contained carbapenemase genes. Of the 18 ertapenem-resistant strains, 17 expressed the efflux pump, and outer membrane protein expression was reduced or absent in 4 strains. Whole-genome sequencing revealed the presence of mutations that introduced premature *ramR* codons stop in 14 strains (77.78%). When a functional copy of *ramR* was restored in the 14 strains, the minimum inhibitory concentration of ertapenem decreased, inhibition of efflux pumps was not detected, and the expression of outer membrane protein OmpK35 was either increased or was restored. These findings reveal the existence of ertapenem-resistant *K. pneumoniae* exhibiting no clonal transmission between strains. Mutations in *ramR* were demonstrated to cause outer membrane protein OmpK35 inhibition and over-expression of efflux pump in some strains, which is implicated in ertapenem resistance only in *K. pneumoniae.*

## Introduction

*Klebsiella pneumoniae* is a gram-negative rod-shaped bacterium that can cause various infections in multiple parts of the body; it is also a major hypervirulent pathogen of hospital-acquired infections (Mukherjee et al., 2021). However, given their widespread use, the resistance rate of *K. pneumoniae* to carbapenems has increased significantly (Lee and Suh, 2021). Ertapenem is a long-acting 1-β-methylcarbapenem, which has a longer serum half-life than meropenem and imipenem (Odenholt, 2001). Results have shown the inhibitory activity of ertapenem against gram-positive, Gram-negative, and anaerobic bacteria *in vitro*. Gram-negative strains producing extended-spectrum β-lactamases (ESBLs) and strains producing AmpC remained still susceptible to ertapenem (Odenholt, 2001). Moreover, As the number ESBL-producing strains increases annually, ertapenem is used as an empirical treatment for community-acquired pneumonia, and abdominal and urinary tract infections (Lob et al., 2018).

The United States Centers for Disease Control and Prevention defines carbapenem-resistant *Klebsiella pneumoniae* (CRKP) as *K. pneumoniae* strains that produce carbapenemase, or are resistant to any carbapenem antibiotics, such as ertapenem, meropenem, imipenem, and doripenem (Guh et al., 2015). Previous studies have shown that the mechanism of drug resistance in CRKP primarily involves the production of various enzymes (ultra–broad-spectrum β lactamase, AmpC enzyme, and carbapenemase), alterations in common binding sites for antibacterial drugs, enhanced expression of efflux pump components, and decreased membrane permeability due to the reduced expression of outer membrane proteins (OMPs) (Tzouvelekis et al., 2012; Padmini et al., 2017). *RamR*, as a local transcriptional regulator, is located upstream of *ramA* and can regulate *ramA* expression to confer a multidrug resistance phenotype (Abouzeed et al., 2008). Previous studies have found that *ramR* mutations are an important factor that mediates the evolution of resistance to tigecycline in *K. pneumoniae* (Moghimi et al., 2021). However, studies on this mutation and the resistance mechanism in *K. pneumoniae* strains that are resistant only to ertapenem are lacking (Mao et al., 2020).

According to the China Antimicrobial Surveillance Network [CHINET] (2021), 12.7, 9.3, and 9.7% of *Enterobacterales* are resistant to ertapenem, imipenem, and meropenem, respectively, among which resistance of ertapenem is higher than meropenem and imipenem. The mechanism underlying ertapenem resistance and meropenem and imipenem susceptibility in *K. pneumoniae* remains unclear. Therefore, the present study collected strains that are resistant to only ertapenem but are sensitive to meropenem and imipenem. The strains were collected from various hospitals in China with the aim of using them to analyze the molecular epidemiological characteristics and mechanism underlying resistance of meropenem- and imipenem-sensitive *K. pneumoniae* strains to ertapenem.

## Materials and methods

### Clinical isolates

A total of 245 strains of CRKP were found in 697 strains of *K. pneumoniae* isolated from 11 hospitals in China between July 2018 and July 2019. *K. pneumoniae* identification was performed using the VITEK^®^ 2 Compact System (bioMérieux, Lyon, France), and 18 strains that were resistant to ertapenem and susceptible to meropenem and imipenem were selected for further analysis. Detailed information about the strains and plasmids used and constructed in this study is shown in Table 1.

**TABLE 1:** Strains and plasmids used in this study.

Strain or plasmid	Description	Source
ATCC35657	Quality control *Klebsiella pneumoniae* strain used for minimum inhibitory concentration testing	Laboratory collection
DH5a	*Escherichia coli*, competent cell strain used for transformation	TIANGEN Biotech
pBad33	Plasmid, a gene replacement vector, apramycin	Laboratory collection
18 strains of *K. pneumoniae*	*K. pneumoniae* resistant to ertapenem but sensitive to meropenem and imipenem	Collected in clinic

### Antimicrobial susceptibility testing

The minimum inhibitory concentrations (MICs) of ertapenem, imipenem, meropenem, tigecycline, polymyxin, cefoxitin, cefepime, cefotaxime, amikacin, chloramphenicol, ceftazidime, piperacillin, ciprofloxacin, aztreonam, piperacillin-tazobactam, and amoxicillin-clavulanic acid were determined using the broth microdilution method. Breakpoints for all the antibiotics were determined following the Clinical and Laboratory Standards Institute guidelines (Clinical and Laboratory Standards Institute, 2018), except for that of tigecycline, which was determined using the U.S. Food and Drug Administration guidelines (Allen, 2016). The MIC of ertapenem was also determined in the presence of the efflux pump inhibitor carbonyl cyanide m-chlorophenylhydrazone (CCCP, 25 mg/L) to investigate the role of the efflux pump in carbapenem resistance (Ni et al., 2016). Carbapenemase phenotype was determined using the EDTA synergistic test and the enzyme inhibitor inhibition test (Liao et al., 2021).

### Screening for the presence of drug resistance genes using polymerase chain reaction

Polymerase chain reaction (PCR) was performed to detect common carbapenem resistance genes (*bla*_*KPC*_, *bla*_*NDM*_, *bla*_*IMP*_, *bla*_*OXA*_-like, *bla*_*DIM*_, *bla*_*SPM*_, *bla*_*VIM*_, *and bla*_*BIC*_), plasmid-mediated AmpC β-lactamase–encoding genes (*bla*_*AAC*_, *bla*_*FOX*_, *bla*_*MOX*_, *bla*_*DHA*_, *bla*_*CIT*_, *and bla*_*EBC*_), and ESBL genes, using previously reported primers (Dallenne et al., 2010). The PCR amplicons were sequenced (Higuchi et al., 1993), and the sequences were compared with those available in the National Center for Biotechnology Information GenBank database using BLAST searches^1^ to confirm the genes detected.

### Multilocus sequence typing and pulsed-field gel electrophoresis

Multilocus sequence typing (MLST) and pulsed-field gel electrophoresis (PFGE) were used to determine the genetic relatedness among the 18 *K. pneumoniae* isolates. PCR and MLST were conducted for seven housekeeping genes (*gapA, infB, mdh, pgi, phoE, rpoB*, and *tonB*), and the sequences were compared using the MLST database (see test footnote 1) to determine the allelic numbers and sequence types (STs). The allelic profiles and STs were assigned using an online database.^2^ For PFGE, bacterial genomic DNA was cleaved with *Xba*I endonuclease (Roche, Penzberg, Germany) and subjected to PFGE using the CHEF-DR^®^ III Variable Angle System (Bio-Rad, Hercules, CA, USA). The PFGE patterns of the 18 strains were compared using BioNumerics software (Applied Maths, Kortrijk, Belgium) with Dice correlation for band matching at a 1.5% position tolerance and the unweighted pair group method with an arithmetic average. Clusters were defined as DNA patterns sharing more than 80% similarity (Neoh et al., 2019).

### Analysis of outer membrane proteins

Outer membrane proteins (OMPs) were isolated and separated using sodium dodecyl sulphate-polyacrylamide gel electrophoresis (SDS-PAGE), as described previously (Carlone et al., 1986). Briefly, the isolated proteins were loaded on a 12% SDS-PAGE and electrophoresed for 25 min at 80 V and 50 min at 150 V (Bio-Rad, Hercules, CA, USA), followed by staining with 1% Coomassie brilliant blue (Beyotime, Shanghai, Beijing, China).

### Whole-genome sequencing and analysis

Deoxyribonucleic acid was extracted using a bacterial genomic DNA extraction kit (TIANGEN, Beijing, China). Sequencing and sequence assembly were performed by Shanghai Yuanxu Biotechnology Co., Ltd. Antimicrobial resistance genes and mutations present throughout the entire genome were analyzed according to the Center for Genomic Epidemiology^3^ guidelines (Chauve et al., 2020).

### Construction and complementation of plasmids carrying *ramR*

To investigate the effect of *ramR* deletion on ertapenem resistance, a plasmid encoding the functional *ramR* was constructed. First, the consensus *ramR* sequence was amplified using primers containing restriction endonuclease sites, and then purified. Second, the amplified fragment and plasmid pBad33, an arabinose-inducible chloramphenicol resistance vector, were double-enzyme–digested to obtain the same cohesive termini, and then ligated overnight with T4 ligase (TaKaRa, D2011A) to generate the plasmid pBad33-*ramR*. Then, these recombinant plasmids were transformed into competent cells *E. coli* DH5a (Novagen, Darmstadt, Germany) *via* chemical conversion in order to obtain more successfully constructed plasmids to complement the wild strains. Successful transformants were identified by selection on Luria-Bertani agar containing 50 mg/L chloramphenicol and confirmed using PCR and sequencing. The primers used to construct pBad33-*ramR* are shown in Table 2.

**TABLE 2:** Primers used in this study.

Primer name	Sequence 5′–3′
*ramR*-*Kpn*I-F	GGTACCGGGTACCCCTGGCACATTTCGTTGAG
*ramR*-*Pst*I-R	CTGCAGGCTGCAGCAAGCAAGCGTTACTGGAA

## Results

### Minimum inhibitory concentrations, antibiotic resistance profiles, and distribution of resistance genes

**FIGURE 1:**
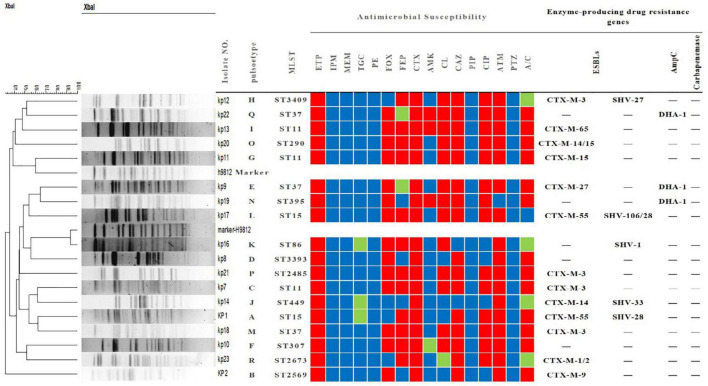
Tree diagram of 18 strains of *Klebsiella pneumoniae* resistant only to ertapenem based on pulse field gel electrophoresis, multilocus sequence typing, characterization, drug sensitivity spectrum and drug resistance gene profile. MLST, multi-locus sequence typing; ETP, ertapenem; IPM, imipenem; MEM, meropenem; TGC, tigecycline; PE, polymyxin; FOX, cefoxitin; FEP, cefepime; CTX, cefotaxime; AMK, amikacin; CL, chloramphenicol; CAZ, ceftazidime; PIP, piperacillin; CIP, ciprofloxacin; ATM, aztreonam; PTZ, piperacillin-tazobactam; A/C, amoxicillin-clavulanic acid. The red, green, and blue squares indicate resistance, intermediate resistance, and susceptibility to each antibiotic, respectively.

In total, 245 out of 697 (35.15%) *K. pneumoniae* strains collected between July 2018 and July 2019 were identified as CRKP strains, of which, 18 (7.4%) were resistant to ertapenem and susceptible to meropenem and imipenem (Figure 1). All 18 isolates were also resistant to cefotaxime and aztreonam and susceptible to polymyxin, piperacillin, and piperacillin-tazobactam. Some of these isolates were also resistant to tigecycline (15/18, 83.3%), cefoxitin (14/18, 77.80%), cefepime (15/18, 83.3%), amicacin (3/18, 16.7%), chloramphenicol (13/18, 72.3%), ceftazidine (16/18, 88.9%), ciprofloxacin (14/18, 77.80%), and amoxicillin clavulanate (13/18, 72.3%) (Figure 1).

**FIGURE 2:**
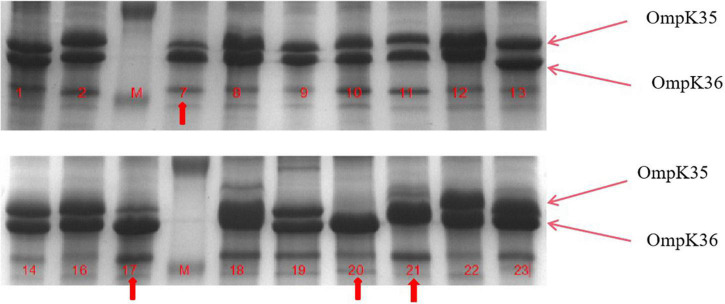
Outer membrane protein (OMP) profiles of *Klebsiella pneumoniae* strains on a 12% sodium dodecyl sulphate-polyacrylamide gel. Lanes 1–4 show the OMPs of strains kp7, kp17, kp20, and kp21. Lanes 4–8 show the OMPs of strains kp7, kp17, kp20, and kp21 functionally supplemented with *ramR*.

Interestingly, carbapenemase genes were not detected in any of the 18 strains, and **mCIM** for carbapenemase were negative. The main sub-types of ESBLs detected in these strains were *bla*_*CTX–M*_ and *bla*_*SHV*_. As shown in Figure 1, three strains (kp22, kp9, kp19) harbored the AmpC-encoding gene *bla*_*DHA–*1_. Strains kp8 and kp10 did not harbor any ESBLs or AmpC-encoding genes (Figure 1). Taken together, these results suggest that ertapenem resistance is mediated by a factor other than carbapenemase. The efflux pump test was positive for 17 of the 18 strains (Figure 1), and OMP expression was reduced or absent in four strains (Figure 2).

### Molecular epidemiology based on multilocus sequence typing and pulsed-field gel electrophoresis

As shown in Figure 1, the 18 strains could be divided into 13 types according to MLST. ST11 and ST37 were the most common types, with three strains each. Two strains belonged to ST15. Meanwhile, the other STs were relatively scattered, with one strain each. PFGE analysis identified 18 different clone groups among the isolates.

### Mechanism of ertapenem resistance

**FIGURE 3:**
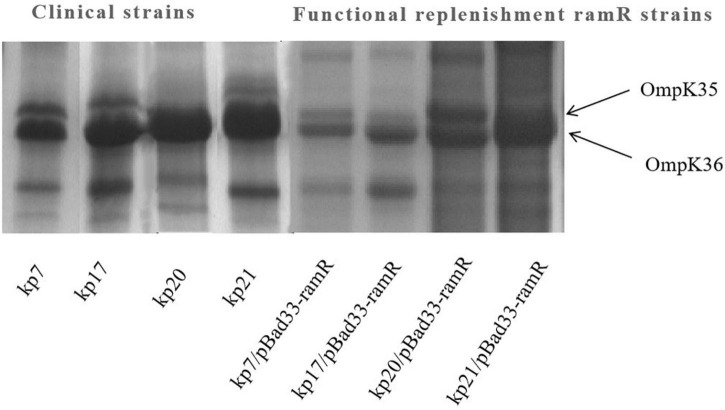
Outer membrane protein (OMP) profiles of Klebsiella pneumoniae strains on a 12% sodium dodecyl sulphate-polyacrylamide gel. Lane 1–4 shows the OMPs of strains kp7, kp17, kp20, and kp21. Lane 4–8 shows the OMPs of strains kp7, kp17, kp20, and kp21 functionally supplemented with ramR.

To further understand the potential drug resistance mechanisms of these strains, we sequenced the 18 strains collected. Surprisingly, 14 strains (77.8%) had early termination mutations in **ramR**, of which 12 strains harbored a termination codon at p.K 194* and two strains a termination codon at p.V 123*. Subsequently, the 14 strains were complemented with a wild-type copy of **ramR**, and found that the MIC of ertapenem decreased by different degrees depending on the strain (Table 3). After inserting **ramR** to the original strain, 11 strains became sensitive to ertapenem, two strains became intermediaries, and one strain was still resistant but its MIC of ertapenem decreased by 4-fold. When the complemented strains were subjected to the efflux pump inhibition test, 10 strains had negative results, whereas all 14 strains had positive results in the previous test, suggesting that efflux pump was the main mechanism mediating the ertapenem resistance (Table 3). Three strains had positive results for the efflux pump inhibition test; however, the expression of OmpK35 was increased, which may indicate that OmpK35 was the main factor in mediating the resistance of these clinical strains to ertapenem (Figures 2, 3). No change in the efflux pump inhibition test results before and after complementation was observed in one strain (kp20), but this strain exhibited recovery of OMP expression (Figures 2, 3).

**TABLE 3:** Drug sensitivity of wild strain and clonal strain.

Isolates. NO.		Clinical strains			Functional replenishment *ramR* strains		
	The change of *ramR*	ETP (MICs, μg/ml)	ETP +CCCP	MICs of ertapenem reduction multiple	ETP (MICs, μg/ml)	ETP +CCCP	MICs of ertapenem reduction multiple
KP12	Termination of p.K194*	4	0.06	66	0.5	0.5	1
KP22	Termination of p.K194*	2	0.5	4	0.5	0.5	1
KP13		8	0.015	534			
KP20	Termination of p.V123*	64	64	1	0.5	0.25	2
KP11		8	2	4			
KP9	Termination of p.K194*	2	0.5	4	0.5	0.25	2
KP19		16	4	4			
KP17	Termination of p.K194*	4	0.5	8	1	0.125	8
KP16	Termination of p.K194*	2	0.015	134	0.125	0.125	1
KP8	Termination of p.K194*	2	0.5	4	0.5	0.5	1
KP21	Termination of p.K194*	8	1	8	0.5	0.125	4
KP7	Termination of p.V123*	8	2	4	2	0.5	4
KP14	Termination of p.K194*	2	0.03	66	0.06	0.06	1
KP1	Termination of p.K194*	2	0.5	4	0.25	0.25	1
KP18	Termination of p.K194*	4	0.5	8	0.5	0.5	1
KP10	Termination of p.K194*	2	0.5	4	1	1	1
KP23	Termination of p.K194*	2	0.125	16	0.5	0.5	1
KP2		2	0.015	134			

The first column is the strain number. The second column is the site of the ramR termination mutation. The third column is the MIC of the ertapenem in clinical strains. The fourth column shows the MIC of ertapenem plus the efflux pump inhibitor CCCP in clinical strains. The fifth column shows the inhibition times of efflux pump inhibition test of clinical strains. The sixth column is the MIC of the ertapenem in clone strains. The seventh column shows the MIC of ertapenem plus the efflux pump inhibitor CCCP in clone strains. The eighth column shows the inhibition times of efflux pump inhibition test of clinical strains.

## Discussion

The mechanism of CRKP resistance is complex, but the treatment and phenotype of CRKP resistant only to ertapenem are different from other carbapenem resistant *K. pneumoniae*. In our study, Eighteen *K. pneumoniae* strains that were resistant only to ertapenem showed no production of carbapenemase and *ampC*. It has been previously documented that efflux pumps and OMPs have been reported to influence produce carbapenemase resistance (Li et al., 2022). Analysis of common drug resistance phenotypes showed that 17 out of the 18 strains had positive results on the efflux pump inhibition test, and four strains showed varying degrees of decreased or even absent OMP expression.

Among the 18 strains of *K. pneumoniae* strains only resistant to ertapenem, 14 strains (77.8%) had a *ramR* termination mutation. These results suggest that the termination mutation in *ramR* is responsible for the main mechanism of ertapenem resistance only in *K. pneumoniae*. As a global transcription factor, *ramR* affects the resistance of various antimicrobial agents by regulating the expression of *ramA* (Abouzeed et al., 2008). Our previous studies have found that termination mutations in *ramR* lead to enhanced expression of efflux pumps, resulting in resistance to a variety of antimicrobial agents (Xu et al., 2021). In the present study, 10 of the 14 strains with *ramR* supplementation changed from positive to negative in efflux pump inhibition test, and ertapenem changed from resistant to sensitive. These data suggest that the termination mutation in *ramR* affects efflux pump expression and leads to ertapenem resistance in non-carbapenemase-producing *K. pneumoniae.* Previous studies have found that OMPs deficiency plays an important role in carbapenem resistance in *Enterobacter aerogens* (Hao et al., 2018). In this study, the other four strains became sensitive to ertapenem after *ramR* supplementation, and the efflux pump did not change, but the OMP expression was enhanced. These data suggest that termination mutations in *ramR* modulate OmpK35 expression to induce ertapenem resistance, the specific major regulatory mechanism of which has been shown in other studies.

This study primarily describes the epidemiological characteristics of ertapenem-resistant CRKP in China. MLST data showed that ST11 and ST37 were dominant clones. PFGE analysis showed that there was no clonal transmission among the 18 strains, and all strains showed sporadic drug resistance. However, these highly pathogenic strains should be closely monitored to prevent their spread. The mechanism of drug resistance was preliminarily explored in the present study, and hence, more specific studies may be required in this context. Furthermore, *ramR* may be the main regulatory target of ertapenem resistance only; therefore, its drug resistance regulatory pathway needs to be elucidated in future studies.

## Data availability statement

The genome sequencing data have been deposited to BioProject accession numbers: PRJNA891454, PRJNA891456, PRJNA891458, PRJNA891462, PRJNA891269, PRJNA891466, PRJNA891468, PRJNA891469, PRJNA891471, PRJNA891507, PRJNA891508, PRJNA891518, PRJNA891517, PRJNA891515, PRJNA891513, PRJNA891511, PRJNA891510, and PRJNA891509 in the NCBI BioProject database.

## Author contributions

MW and YY designed the research and wrote the manuscript. DW, TH, DL, WY, and JF performed the experiments. LZ and DZ did data analysis. All authors contributed to the article and approved the submitted version.

## Footnotes

1
https://bigsdb.pasteur.fr/index.html

2
https://pubmlst.org/

3
http://www.genomicepidemiology.org/
