# Corrigendum: Molecular epidemiology and mechanism of *Klebsiella pneumoniae* resistance to ertapenem but not to other carbapenems in China

**DOI:** 10.3389/fmicb.2023.1185340

**Published:** 2023-04-06

**Authors:** Dongliang Wang, Minggui Wang, Tianpeng He, Dan Li, Liqin Zhang, Dongquan Zhang, Junshuai Feng, Wenli Yang, Yuan Yuan

**Affiliations:** ^1^Department of Critical Care Medicine, Gansu Provincial Hospital, Lanzhou, Gansu, China; ^2^Institute of Antibiotics, Huashan Hospital, Fudan University, Shanghai, China; ^3^Key Laboratory of Clinical Pharmacology of Antibiotics, National Health Commission of the People's Republic of China, Shanghai, China

**Keywords:** ertapenem resistance, *Klebsiella pneumoniae*, *ramR*, efflux pump, outer membrane protein

In the published article, there was an error. In the last sentence of the abstract, the expression of the outer membrane protein OmpK35 and efflux pump gene was incorrectly stated.

The sentence previously stated: “Mutations in *ramR* were demonstrated to cause efflux pump inhibition and over-expression of outer membrane protein OmpK35 in some strains, which is implicated in ertapenem resistance only in *K. pneumoniae*.”

The corrected sentence is: “Mutations in *ramR* were demonstrated to cause outer membrane protein OmpK35 inhibition and over-expression of efflux pump in some strains, which is implicated in ertapenem resistance only in *K. pneumoniae*.”

The authors apologize for this error and state that this does not change the scientific conclusions of the article in any way. The original article has been updated.

## Publisher's note

All claims expressed in this article are solely those of the authors and do not necessarily represent those of their affiliated organizations, or those of the publisher, the editors and the reviewers. Any product that may be evaluated in this article, or claim that may be made by its manufacturer, is not guaranteed or endorsed by the publisher.

