# Supplementary material for: Molecular epidemiology and mechanism of Klebsiella pneumoniae resistance to ertapenem but not to other carbapenems in China
Source: Front Microbiol. 2022 Nov 8;13:974990. doi: 10.3389/fmicb.2022.974990 (PMC9678918; doi:10.3389/fmicb.2022.974990)
Supplement: Supplementary file 1 [file Presentation_1.PPTX]

## Slide 1
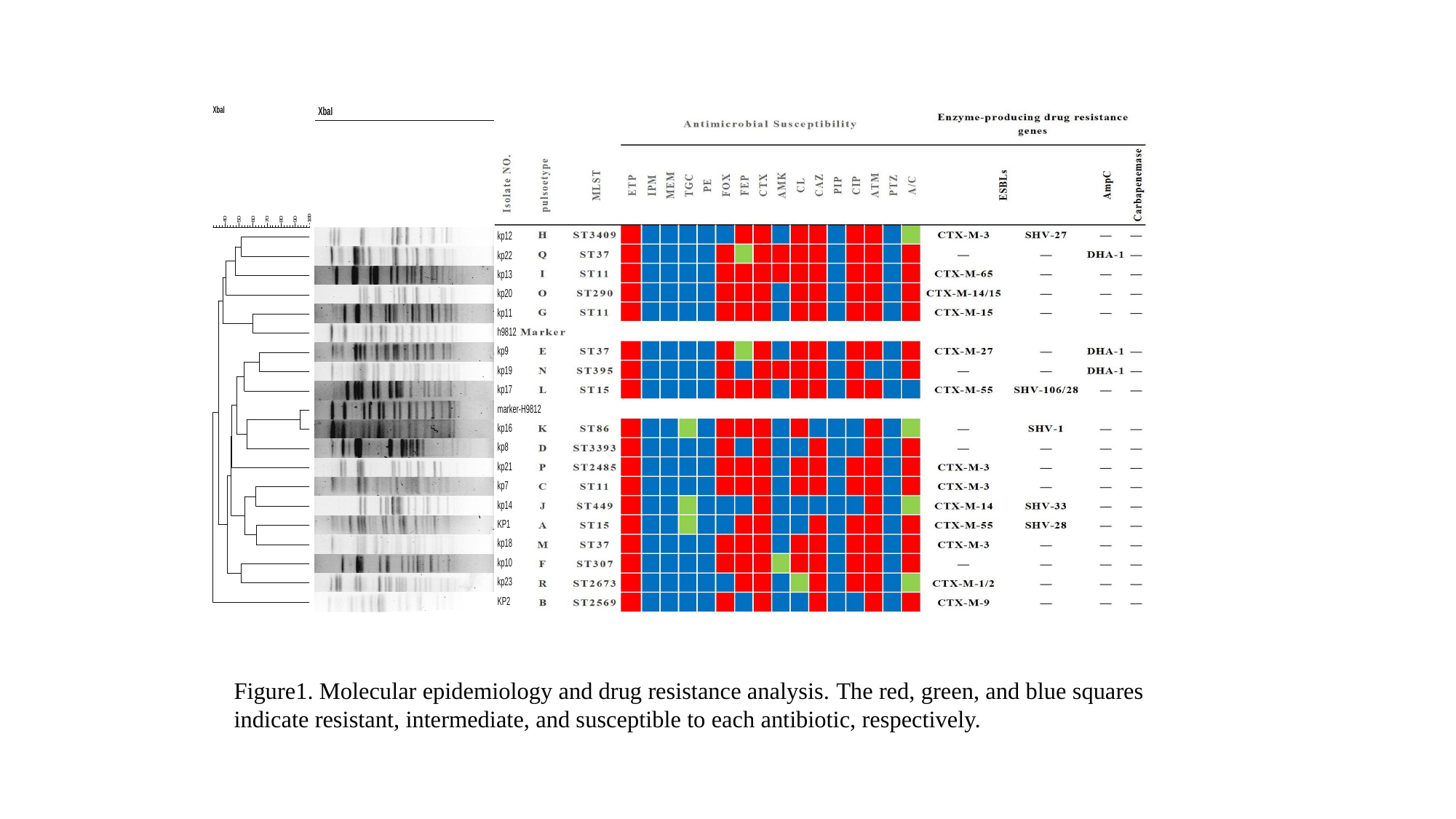

Figure1. Molecular epidemiology and drug resistance analysis. The red, green, and blue squares indicate resistant, intermediate, and susceptible to each antibiotic, respectively.

## Slide 2
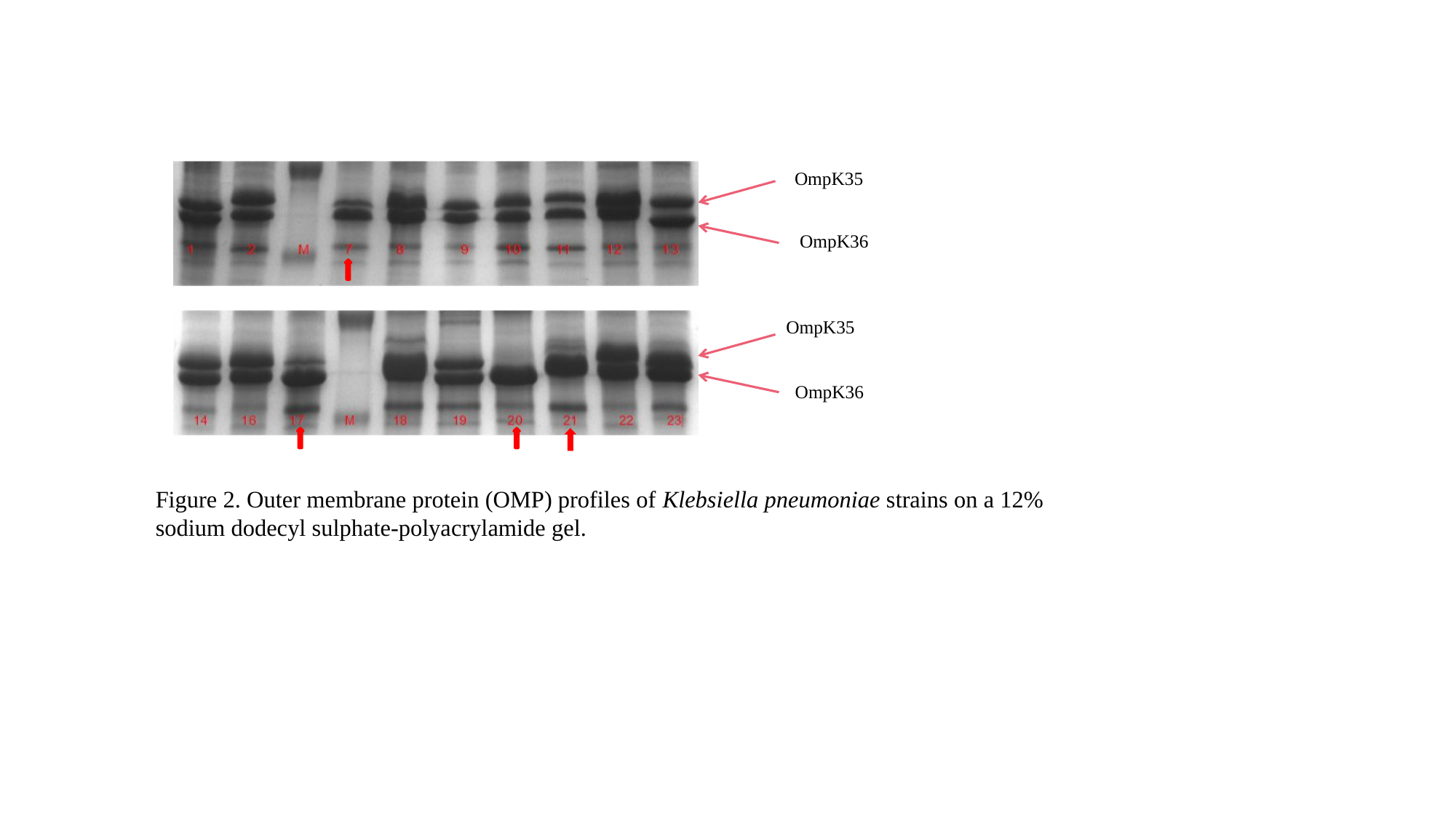

OmpK35
OmpK36
OmpK35
OmpK36
Figure 2. Outer membrane protein (OMP) profiles of Klebsiella pneumoniae strains on a 12% sodium dodecyl sulphate-polyacrylamide gel.

## Slide 3
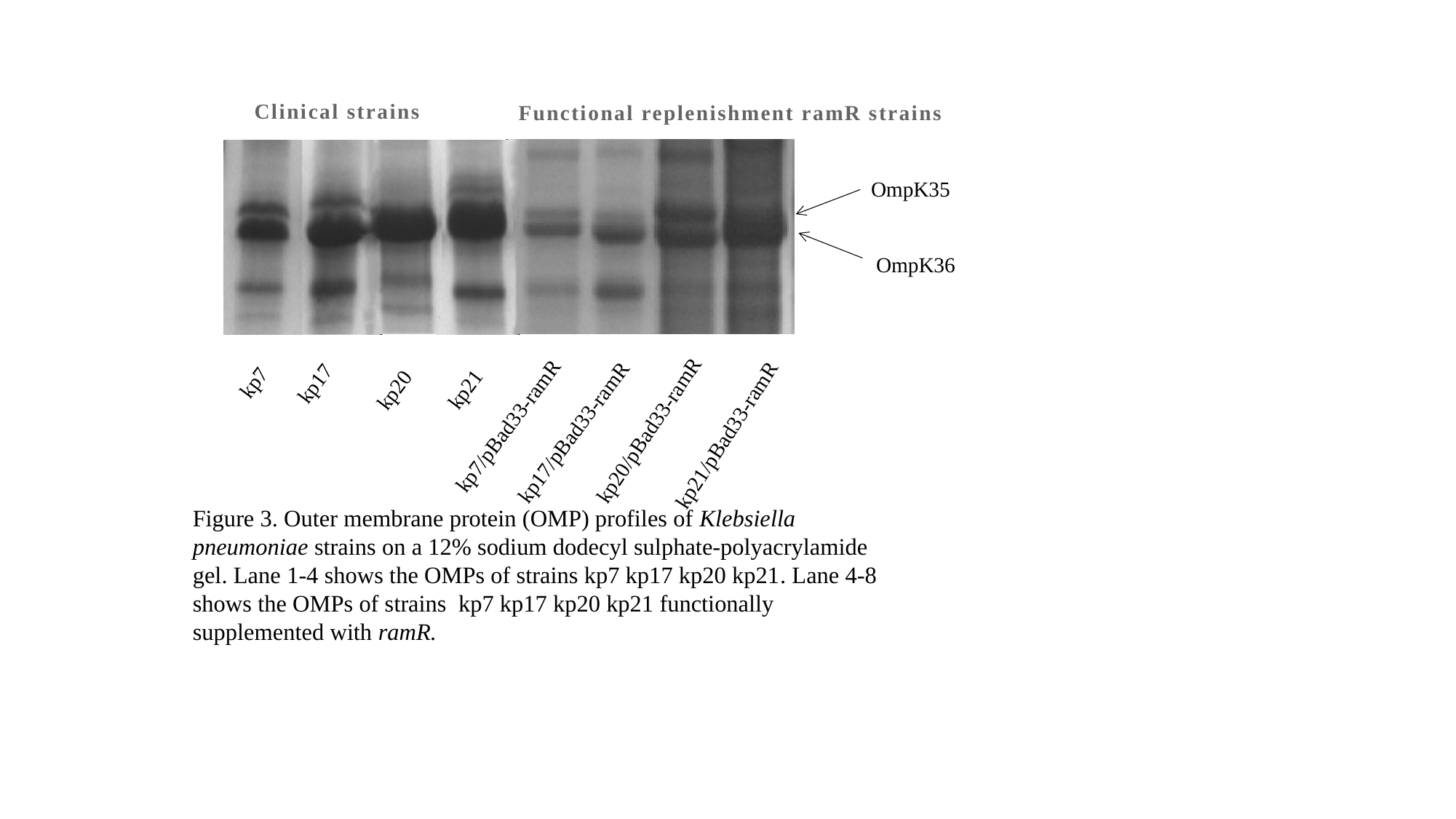

Clinical strains
Functional replenishment ramR strains
OmpK35
OmpK36
kp7
kp17
kp20
kp21
kp7/pBad33-ramR
kp20/pBad33-ramR
kp17/pBad33-ramR
kp21/pBad33-ramR
Figure 3. Outer membrane protein (OMP) profiles of Klebsiella pneumoniae strains on a 12% sodium dodecyl sulphate-polyacrylamide gel. Lane 1-4 shows the OMPs of strains kp7 kp17 kp20 kp21. Lane 4-8 shows the OMPs of strains kp7 kp17 kp20 kp21 functionally supplemented with ramR.
